# Endometriosis and Ovarian Cancer: Insights from NHANES and Mendelian Randomization Analysis

**DOI:** 10.1007/s43032-025-01910-x

**Published:** 2025-06-23

**Authors:** Dong Liu, Yuke Wu, Kunyan Zhou

**Affiliations:** 1 Department of Obstetrics and Gynecology, West China Second University Hospital, Sichuan University, Chengdu, China; 2 Key Laboratory of Birth Defects and Related Diseases of Women and Children (Sichuan University), Ministry of Education, Chengdu, China

**Keywords:** Endometriosis, Cancer, Mendelian randomization, Ovarian cancer, Epidemiology

## Abstract

Endometriosis describes the growth of extra-uterine endometrium, causing pain and inflammation, and the condition has been estimated to affect 10% of women of reproductive age. It remains under-diagnosed and has been linked to increased cancer risk. This study evaluated the correlation between endometriosis and cancer. The significance of specific loci to cancer was analyzed via Mendelian randomization (MR). Of the 4092 samples included, cancer diagnoses were self-reported by 326 women with endometriosis and 3766 women without endometriosis. Correlations between individual cancer types and endometriosis were analyzed by multivariable logistic regression models and odds ratios (OR) adjusted for confounders. Mortality was evaluated by Kaplan–Meier survival analysis. Genetic variants associated with endometriosis were linked to ovarian cancer by MR analysis. Women with endometriosis had a higher association of overall cancer (OR = 1.80, 95% CI: 1.19—2.72) and this was especially the case for ovarian cancer (OR = 11.40, 95% CI: 3.00—43.34). Subgroup and sensitivity analyses confirmed the robustness of these results. Kaplan–Meier analysis indicated lower survival from all causes in women with endometriosis (p = 0.022) but no significant difference in cancer survival. MR analysis supported a positive relationship between endometriosis and ovarian cancer (OR = 1.203, 95% CI: 1.011—1.433). The history of endometriosis was positively associated with the history of overall and ovarian cancer. Women with a history of endometriosis should have access to enhanced cancer surveillance and proactive management. Further research is needed to confirm these associations and to give mechanistic insights.

**Graphical Abstract:**

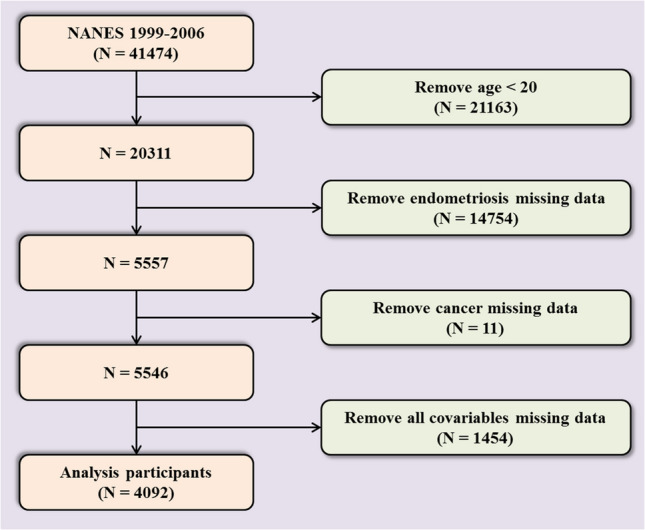

**Supplementary Information:**

The online version contains supplementary material available at 10.1007/s43032-025-01910-x.

## Background

Endometriosis is an incurable, under-diagnosed, systemic inflammatory disease affecting millions world-wide [1]. It is a persistent gynecological condition resulting from the growth of endometrial-like tissue beyond the confines of the uterus and causes inflammation, dysmenorrhea, pelvic discomfort and infertility [2]. The condition affects around 10% of women of reproductive age with an impact on quality of life and a significant socio-economic burden due to the chronic nature [2, 3]. It appears that genetic, environmental, hormonal and immune factors all contribute to the pathogenesis [3, 4]. Endometriosis is usually considered to be a non-malignant condition but has features reminiscent of cancer, such as the formation of both local and distant lesions and the infiltration of surrounding tissues which may impair organ function [5]. ​Diagnosing endometriosis presents multifaceted challenges, resulting in delays and misdiagnoses, emphasizing the need for timely and accurate identification. Delayed diagnosis often leads to prolonged suffering, reduced quality of life and increased health-care costs [6, 7] whereas early detection allows for more appropriate pain relief, fertility preservation and disease monitoring [8–10]. Although endometriosis is incurable, it can be managed through various long-term treatment strategies to alleviate symptoms and improve fertility [11]. Common treatments include pharmacological therapies such as oral contraceptives, progestins, and GnRH agonists to alleviate symptoms and slow disease progression [12]. In severe cases, surgical options, such as excision of lesions or hysterectomy, may be effective treatment choices [13].

Around 20% of ovarian and deep endometriosis lesions harbor somatic mutations associated with cancer development, indicating a potential association between endometriosis and oncogenesis [14, 15]. Such mutations are present in healthy, eutopic endometrium but occur at significantly higher rates in women with endometriosis [16]. Indeed, systematic reviews have shown that endometriosis is associated with higher summary relative risks for ovarian cancer and thyroid cancer, a minimally increased risk of breast cancer (4%) and a reduced risk of cervical cancer [17]. However, other studies have found no significant association between endometriosis and risks of breast cancer, melanoma and non-Hodgkin lymphoma [18, 19]. Therefore, the recognition of endometriosis as a potential risk factor for cancer is justified but most studies have had small sample sizes, diverse study populations, short follow-up durations and inadequate adjustment for confounders [6, 8, 14–17]. Such limitations have led to inconsistent results, varying levels of risk bias and significant heterogeneity across studies. Moreover, the observational nature of most studies makes it difficult to establish causal relationships between endometriosis and cancer risk [1, 14, 15]. There is thus a requirement for robust, large-scale investigations to confirm the preliminary pre-existing work.

Mendelian randomization (MR) involves the use of genetic variants as instrumental variables to assess the association of a modifiable exposure with an outcome and mitigates the drawbacks of conventional observational studies by minimizing biases caused by confounding and reverse causality [20]. The current study used the National Health and Nutrition Examination Survey (NHANES) dataset from 1999 to 2006 to investigate the prevalence of overall cancer and of individual cancer types in patients with endometriosis compared with the general population. Potential confounders were considered, including age, race, comorbidities and lifestyle factors. MR was used as an approach to investigate associations between endometriosis and ovarian cancer. The use of a large-scale dataset was an approach intended to mitigate the constraints of previous observational studies. We hope to use large-scale datasets to assess the correlation between endometriosis and cancer history.

## Methods

### Study Population

The NHANES study employs a complex, multistage probability survey design to ensure national representativeness in cross-sectional data collection. Data has been collated by the National Center for Health Statistics (NCHS). The data sets utilized during the current study are available on the NHANES website (https://wwwn.cdc.gov/nchs/nhanes/default.aspx). Data encompassing information on demographics, dietary intake, examination results, laboratory results and questionnaire responses. The NHANES survey includes different samples of participants each year. For those participants who signed the follow-up consent form, their mortality data was recorded in the National Death Index (NDI) and linked to the NHANES dataset. This study included female participants with age ≥ 20 years, and completed the reproductive health questionnaire from four NHANES cycles (1999 to 2006). This study excluded participants under 20 years; had missing data on diagnosis of endometriosis and cancer; missing data on any covariables.

### Definitions of Endometriosis

Endometriosis status was self-reported by a yes/no response to the question “HHQ360: Has a doctor or other health professional ever told you that you have endometriosis?”.

### Definitions of Cancer

Cancer status was self-reported by a yes/no response to the question “MCQ220: Has a doctor or other health professional ever told you had cancer or malignancy?”. Cancer types were identified by the question “MCQ230a: what kind of cancer?”, and were also self-reported. If the number of occurrences of a specific type of cancer was fewer than five, it was categorized as “other cancer” along with cancers that did not fit into any category.

### Covariates

Potential confounders, such as the demographic characteristics of age, race, education level (high school/high school or above), marital status and poverty income ratio (PIR); physical examination results, such as body mass index (BMI); lifestyle factors, such as smoking and drinking status; comorbidities, such as diabetes mellitus (DM) and hypertension, were used as covariates. Race was divided into White non-Hispanic, Mexican American, Black non-Hispanic, Other Hispanic and Other Race (including Multi-Racial). Smoking status was divided into “yes” (having smoked over 100 cigarettes during lifetime) or “no” (having smoked fewer than 100 cigarettes during lifetime) [21]. Drinking was categorized as “yes” (having had more than 12 drinks during lifetime) or “no” (having had fewer than 12 drinks during lifetime) [21].

DM was defined as meeting any of the following criteria: (1) a self-reported history of diabetes, (2) ≥ 6.5% glycosylated hemoglobin levels, (3) current use of glucose-lowering medication or insulin therapy, (4) random blood glucose measurements of ≥ 11.1 mmol/L, (5) blood glucose levels of ≥ 11.1 mmol/L following a 2 h oral glucose tolerance test or (6) fasting blood glucose levels of ≥ 7.0 mmol/L [22].

Hypertension was defined as meeting any of the following criteria: (1) the current use of antihypertensive medication, (2) a physician's diagnosis of hypertension or (3) average systolic blood pressure readings of ≥ 140 mmHg or average diastolic blood pressure readings of ≥ 90 mmHg [23].

### Identification of Mortality

Date of death was determined by cross-referencing data from the NCHS and the NDI up to December 31, 2019. Cause of death was categorized according to the International Statistical Classification of Diseases, 10th Revision (ICD-10), with cancer mortality specifically defined as deaths resulting from malignant neoplasms (ICD-10 codes C00-C97). Since only participants who signed the informed consent form for mortality follow-up have their death information recorded and linked to the NHANES data, the number of deaths obtained in this study does not represent the actual total number of deaths among all participants. However, it still provides valuable reference information.

### MR

Instrumental variables were single nucleotide polymorphisms (SNPs) from the Genome-Wide Association Study (GWAS) dataset supplied by FinnGen Research (https://r10.finngen.fi/pheno/N14_ENDOMET_INFERT) to enable investigation of a relationship between endometriosis and ovarian cancer. Endometriosis was diagnosed according to ICD-9 and ICD-10 criteria and the GWAS dataset included 19,339,618 loci variations from 3,575 cases and 219,470 non-endometriosis participants. Outcome GWAS statistics for ovarian cancer were obtained from the UK Biobank (https://pheweb.org/UKB-SAIGE/pheno/184.11), comprising 391,798 loci variations from 2,103 cases and 389,695 participants without ovarian cancer [24].

The significance threshold for IV selection was p < 5 × 10 ^−8^. SNPs were pruned by a clumping procedure with an R^2 < 0.01 and a clumping distance of 10,000 kb to avoid bias due to linkage disequilibrium. F statistics were used to assess IV robustness with F > 10 indicating a robust IV which could be included in the analysis to minimize the impact of weak instrument bias [25]. MR methods used were inverse-variance weighted (IVW), MR Egger, weighted median, weighted mode and MR-PRESSO [26]. The IVW method is often regarded as the most powerful [20]. A value of p < 0.05 was considered to indicate statistical significance.

Heterogeneity was evaluated using Cochrane’s Q statistic and p > 0.05 indicated a lack of significant heterogeneity. MR Egger and MR-PRESSO tests were used to assess horizontal pleiotropy and identify outliers. MR Egger regression was used to detect horizontal pleiotropy with p > 0.05 signifying its absence [27]. Leave-one-out sensitivity analysis was conducted to identify outliers and validate robustness.

### Statistical Analysis

Survey weights supplied by the NHANES website (https://wwwn.cdc.gov/Nchs/Nhanes/1999-2000/DEMO.htm#WTINT2YR. ) were appropriately utilized. Continuous covariate variables are presented as means ± standard error and differences were evaluated by Student’s t test. Categorical variables are represented as percentages and were assessed using Chi-square (χ2) or Fisher's exact tests. Multivariable logistic regression models were employed to ascertain the association between endometriosis and cancer, producing odds ratios and corresponding 95% confidence intervals. Three models of logistic regression were used: 1) Crude Model: no adjustment for confounders; Model 1: adjusted for age and race which are significant risk factors for cancer; Model 2: adjusted for age, race, marital status, PIR, education, BMI, smoking, drinking, hypertension and DM to minimize the impact of confounding factors [28, 29].

Subgroup analyses and interaction tests were performed to assess the association between endometriosis and cancer in various populations by stratifying according to age: < 30; 30—50; > 50; BMI: < 25; 25—30; > 30; educational status; smoking; drinking; DM and hypertension. Analyses were adjusted for all covariates except the stratifying factor. Kaplan–Meier analysis was conducted to assess correlations between overall mortality and cancer-specific mortality relative to endometriosis while controlling for all covariates specific on the population selected for this study.

Sensitivity analyses were performed through the imputation of missing values, followed by logistic regression analyses adjusted for all covariates. Categorical variables with missing data were designated as a missing indicator category (educational status: n = 5; marital status: *n* = 152; smoking status: *n* = 3; DM: *n* = 1,012; hypertension: *n* = 1; drinking status: *n* = 2). Continuous variables with missing data were imputed using the mean (BMI: *n* = 57; PIR: *n* = 359).

MR and all statistical analyses were conducted utilizing the “nhanesR” and “TwoSampleMR” packages in R software (Version 4.3.3). Statistical significance was determined as p < 0.05.

## Results

### Population Characteristics

**Fig. 1:**
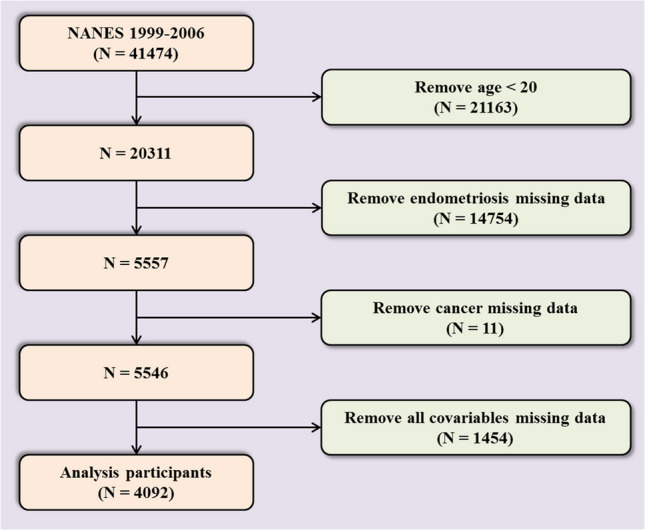
Flow chart of participant selection

A total of 41,474 participants were identified from the NHANES 1999–2006 dataset and application of inclusion and exclusion criteria allowed 4092 participants to be enrolled, 326 with and 3,766 without endometriosis (Fig. 1). Clinical characteristics are summarized in Table 1. The cohort was aged between 20 and 54 years. Age (40.87 ± 0.39 vs. 37.21 ± 0.20) and PIR (3.29 ± 0.11 vs. 2.95 ± 0.05) were higher for endometriosis patients than non-endometriosis patients (all p < 0.0001). BMI, DM and drinking status showed no significant differences. Race, marital status, educational status, smoking status, drinking status, hypertension and cancer showed statistically significantly differences between participants with endometriosis and those without. The study population was predominantly non-Hispanic White, comprising 47.51% of participants, and they accounted for 69.02% of endometriosis cases. In comparison, non-Hispanic BlacksWhite Mexican Americans, and Hispanics make up 22.29%, 21.60%, and 4.59% of the total population, respectively, but their representation in endometriosis cases was lower, at 18.10%, 8.28%, and 1.84% These findings suggest that non-Hispanic Whites had a higher likelihood of developing endometriosis compared to other ethnic groups (Table 1).

**Table 1:** Characteristics of study participants

Variable	Total	No endometriosis (*n* = 3766)	Endometriosis (*n* = 326)	*p* value
Age, years	38.069 ± 0.201	37.740 ± 0.216	41.108 ± 0.387	< 0.0001
PIR	2.998 ± 0.044	2.967 ± 0.048	3.281 ± 0.106	0.009
BMI, kg/m^2^	28.128 ± 0.181	28.133 ± 0.189	28.083 ± 0.427	0.91
Race, n (%)				< 0.0001
White	1944 (47.51)	1719 (45.65)	225 (69.02)	
Black	912 (22.29)	853 (22.65)	59 (18.10)	
Mexican American	884 (21.60)	857 (22.76)	27 (8.28)	
Hispanic	188 (4.59)	182 (4.83)	6 (1.84)	
Other race	164 (4.01)	155 (4.12)	9 (2.76)	
Marital, n (%)				< 0.001
Married	2495 (60.97)	2285 (60.67)	210 (64.42)	
Divorced	716 (17.50)	639 (16.97)	77 (23.62)	
Never married	881 (21.53)	842 (22.36)	39 (11.96)	
Education, n (%)				0.013
High school or above	3177 (77.64)	2892 (76.79)	285 (87.42)	
Under high school	915 (22.36)	874 (23.21)	41 (12.58)	
Smoking, n (%)				0.018
No	2484 (60.70)	2315 (61.47)	169 (51.84)	
Yes	1608 (39.30)	1451 (42.117)	157 (49.753)	
Drinking, n (%)				0.058
No	685 (16.74)	651 (17.29)	34 (10.43)	
Yes	3407 (83.26)	3115 (82.71)	292 (89.57)	
DM, n (%)				0.983
No	3712 (90.71)	3416 (90.71)	296 (90.79)	
Yes	380 (9.29)	350 (9.29)	30 (9.20)	
Hypertension, n (%)				0.008
No	3123 (76.39)	2909 (77.24)	214 (96.32)	
Yes	969 (23.68)	857 (22.76)	112 (34.36)	
Age, n (%)				< 0.0001
< 30	1105 (27.00)	1070 (28.41)	35 (10.74)	
30—50	2521 (61.61)	2285 (60.67)	236 (72.39)	
> 50	466 (11.39)	411 (10.91)	55 (16.87)	
BMI_, n (%)				0.686
< 25	1482 (36.22)	1360 (36.11)	122 (37.42)	
25—30	1093 (26.71)	1001 (26.58)	92 (28.22)	
> 30	1517 (37.07)	1405 (37.31)	112 (34.36)	
Cancer, n (%)				< 0.0001
No	3881 (94.84)	3595 (95.46)	286 (87.73)	
Yes	211 (5.16)	171 (4.54)	40 (12.27)	
Breast cancer, n (%)				0.61
No	4058 (99.17)	3736 (99.20)	322 (98.77)	
Yes	34 (0.83)	30 (0.80)	4 (1.23)	
Cervix cancer, n (%)				
No	4024 (98.34)	3710 (98.51)	314 (96.32)	< 0.01
Yes	68 (1.66)	56 (1.49)	12 (3.68)	
Uterus cancer, n (%)				0.70
No	4078 (99.66)	3754 (99.68)	324 (99.39)	
Yes	14 (0.34)	12 (0.32)	2 (0.61)	
Ovarian cancer, n (%)				< 0.01
No	4080 (99.71)	3758 (99.79)	322 (98.77)	
Yes	12 (0.29)	8 (0.21)	4 (1.23)	
Thyroid cancer, n (%)				1.00
No	4082 (99.76)	3757 (99.76)	325 (99.69)	
Yes	10 (0.24)	9 (0.24)	1 (0.31)	
Skin cancer, n (%)				0.04
No	4061 (99.24)	3741 (99.34)	320 (98.16)	
YES	31 (0.76)	25 (0.66)	6 (1.84)	
Blood cancer, n (%)				< 0.01
No	4085 (99.83)	3762 (99.89)	323 (99.08)	
Yes	7 (0.17)	4 (0.11)	3 (0.92)	
Other cancer, n (%)				< 0.001
No	4057 (99.14)	3739 (99.28)	318 (97.55)	
Yes	35 (0.85)	27 (0.72)	8 (2.45)	

Weighted data from NHANES, 1999—2006. Total number of participants, *n* = 4092

Mean ± SE presented for continuous variables;

*n* (%) presented for categorical variables. BMI: body mass index;

*PIR* poverty income ratio; *DM* diabetes mellitus

### Association of Endometriosis with Cancer

211 participants developed cancer and logistical regression analysis was conducted to indicate any correlation of breast (*n* = 34); cervical (*n* = 68); uterine (*n* = 14); ovarian (*n* = 12); thyroid (*n* = 10); skin (*n* = 31) blood (*n* = 7) and other cancers (*n* = 35) with endometriosis (Table 2).

**Table 2:** Weighted multivariable logistic regression analysis of the association between endometriosis and various types of cancer

Cancer	*n*	Crude model		Model 1		Model 2	
Type		OR (95%CI)	*p* value	OR (95%CI)	*p* value	OR (95%CI)	*p* value
Total cancer	211	2.28 (1.52, 3.41)	< 0.001	1.84 (1.23, 2.76)	0.004	1.80 (1.19, 2.72)	0.01
Breast	34	1.25 (0.36, 4.36)	0.72	0.95 (0.27, 3.31)	0.93	0.99 (0.28, 3.51)	0.98
Cervix	68	1.76 (0.89, 3.47)	0.10	1.61 (0.80, 3.26)	0.18	1.44 (0.70, 2.96)	0.31
Uterus	14	1.02 (0.30, 3.48)	0.97	1.03 (0.30, 3.54)	0.96	1.08 (0.31, 3.73)	0.91
Ovary	12	7.81 (1.81, 33.75)	0.01	9.32 (2.38, 36.49)	0.002	11.40 (3.00, 3.34)	< 0.001
Thyroid	10	1.32 (0.14, 12.69)	0.81	1.03 (0.11, 9.88)	0.98	1.08 (0.11, 10.83)	0.94
Skin	31	2.37 (0.90, 6.25)	0.08	1.75 (0.65, 4.72)	0.27	1.72 (0.62, 4.77)	0.29
Blood	7	4.12 (0.82, 20.58)	0.08	3.13 (0.60, 16.30)	0.17	3.24 (0.55, 19.04)	0.19
Other	35	4.02 (1.62, 9.97)	0.003	3.22 (1.28, 8.10)	0.01	3.04 (1.14, 8.07)	0.03

Crude model: no adjustment;

Model 1: adjusted for age and race;

Model 2: adjusted for age, race, PIR, BMI, marital status, educational status, smoking, drinking, DM, hypertension

*OR* odds ratios; *CI* confidence interval

Crude model analysis showed positive association between endometriosis and cancer (OR = 2.28, 95% CI: 1.52—3.41, p < 0.001) with model 1 also showing a significant, although attenuated, association (OR = 1.84, 95% CI: 1.23—2.76, p = 0.004) after adjustment for age and race. Use of model 2 with adjustment for PIR, BMI, marital status, educational status, smoking, drinking, diabetes and hypertension did not substantially change this association (OR = 1.80, 95% CI: 1.19—2.72, p = 0.01).

The association between endometriosis and ovarian cancer remained significant across all models, giving an OR of 7.81 (95% CI: 1.81—33.75, p = 0.01) when the crude model was used, 9.32 (95% CI: 2.38—36.49, p = 0.002) when Model 1was used and 11.40 (95% CI: 3.00—43.34, p < 0.001) with model 2. An enduring relationship between endometriosis and ovarian cancer was thus demonstrated and this was independent of potential confounding factors. No significant associations were found between endometriosis and breast, cervical, uterine, thyroid, skin or blood cancers. However, endometriosis did show an association with other cancers, a group which included examples of cancer types with fewer than 5 patients (stomach (n = 2), colon (n = 2), rectum (n = 1), liver (n = 2), brain (n = 1), lung (*n* = 3), bone (*n* = 2), kidney (*n* = 2), oral (*n* = 2), soft tissue (*n* = 2)) and cancers for which no category was given (*n* = 16). Crude model analysis gave an OR = 4.02 (95% CI: 1.62—9.97, p = 0.003) and this association remained significant after full adjustment in Model 2 with an OR = 3.04 (95% CI: 1.14—8.07, p = 0.03).

### Subgroup Analyses

Subgroup analyses illuminated associations between endometriosis and cancer, stratified by age, BMI, educational status, drinking, smoking, DM and hypertension. Interaction terms for each stratified factor were found to be non-significant, indicating consistent associations across subgroups.

A significant association was observed in the 30—50 age group (OR = 2.088, 95% CI: 1.332 −3.273, p = 0.002) and in individuals with a BMI between 25 and 30 (OR = 2.376, 95% CI: 1.069—5.277, p = 0.034). There was also a significant association in those of higher educational status (OR = 1.914, 95% CI: 1.232—2.976, p = 0.005). Endometriosis was also associated with cancer among drinkers (OR = 1.833, 95% CI: 1.208—2.782, p = 0.005) and non-smokers (OR = 2.327, 95% CI: 1.225—4.420, p = 0.011), in those without DM (OR = 1.882, 95% CI: 1.250—2.832, p = 0.003) and those without hypertension (OR = 1.807, 95% CI: 1.110—2.941, p = 0.018). Thus, considerable impact of demographics and lifestyle factors was found (Table 3).

**Table 3:** Subgroup analyses on the effect of interaction between the covariates and participants with or without history of endometriosis

Characteristic	Adjusted model OR (95%CI)	*p* value	p for interaction
Age			0.255
< 30	1.339 (0.160, 11.228)	0.783	
30—50	2.088 (1.332, 3.273)	0.002	
> 50	1.039 (0.427, 2.525)	0.931	
BMI			0.834
< 25	1.442 (0.730, 2.851)	0.285	
25—30	2.376 (1.069, 5.277)	0.034	
> 30	1.863 (0.874, 3.969)	0.105	
Educational status			0.39
High school or above	1.914 (1.232, 2.976)	0.005	
Up to high school	0.838 (0.152, 4.631)	0.836	
Drinking			0.574
No	1.150 (0.097, 13.625)	0.910	
Yes	1.833 (1.208, 2.782)	0.005	
Smoking			0.357
No	2.327 (1.225, 4.420)	0.011	
Yes	1.542 (0.921, 2.580)	0.097	
DM			0.588
No	1.882 (1.250, 2.832)	0.003	
Yes	1.380 (0.327, 5.824)	0.654	
Hypertension			0.776
No	1.807 (1.110, 2.941)	0.018	
Yes	1.748 (0.820, 3.727)	0.144	

Adjusted for covariates, age, race, marital status, PIR, educational status, BMI, smoking, drinking, hypertension and DM, with the exception of the stratified factor itself

*OR* odds ratio; *CI* confidence interval; *BMI* body mass index; *DM* diabetes mellitus

### Kaplan–Meier Analysis

**Fig. 2:**
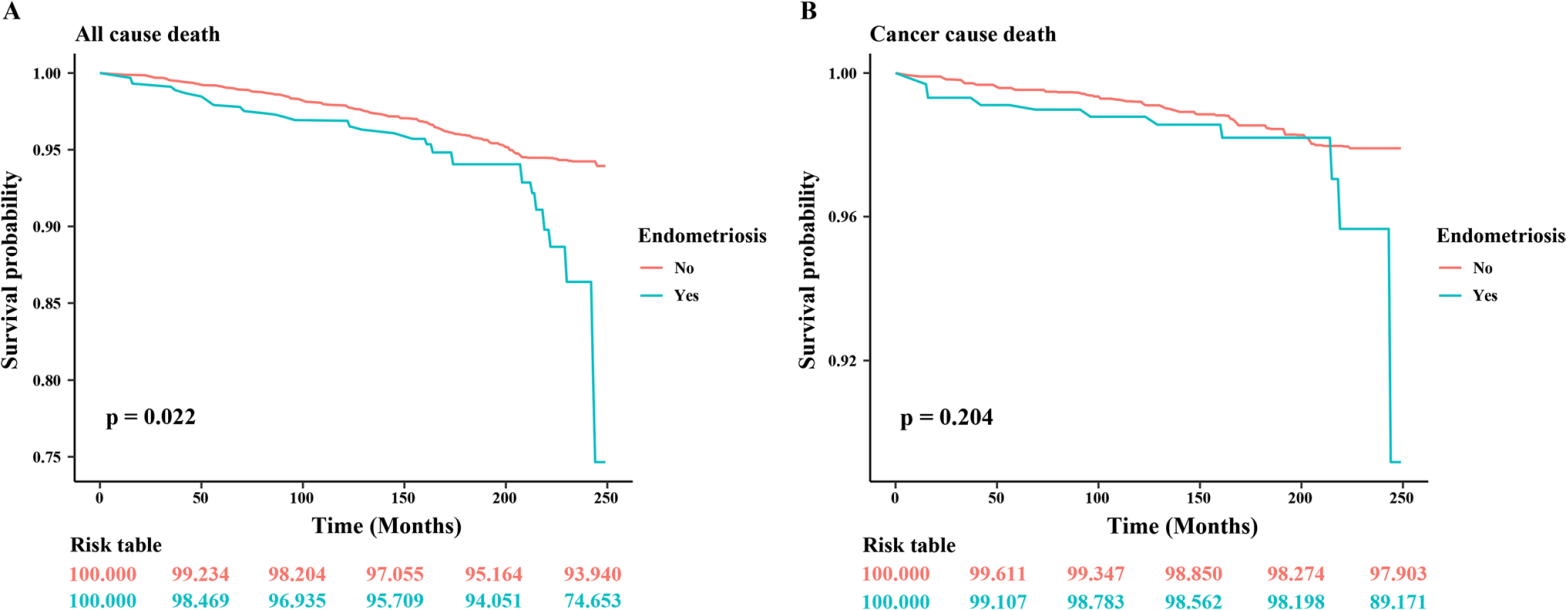
Kaplan–Meier survival curves of all-cause (**A**) and cancer (**B**) mortality in individuals with and without endometriosis. Risk tables indicate the proportion of participants at risk at different time points

A total of 225 all-cause deaths and 76 cancer-specific deaths were documented for the total of 4092 participants. Endometriosis patients had a significantly lower survival from all causes at 74.653% compared with non-endometriosis patients (93.940%, p = 0.022). Mortality increased sharply during the later part of the observation period, after 165 months, for endometriosis patients but not for non-endometriosis patients (Fig. 2A-B). By contrast, cancer-specific mortality did not differ between endometriosis patients and non-endometriosis patients (p = 0.204), although there was a non-significant trend towards lower cancer survival by the former group (89.171% vs 97.903%). These values imply that endometriosis was associated with greater susceptibility to non-cancer mortality, although the trend for cancer-specific death warrants further investigation.

### Sensitivity Analysis

A total of 5546 participants, 379 with and 5167 without endometriosis, were included in the sensitivity analysis after supplementation of missing values (as described above). The crude model showed an OR of 2.31 (95% CI: 1.55—3.43, p < 0.0001) for endometriosis impact on total cancer which remained significant after adjusting for age and race in Model 1 (OR: 1.81, 95% CI: 1.22—2.71, p = 0.004) and further adjusting for additional confounders in Model 2 (OR: 1.73, 95% CI: 1.13—2.64, p = 0.01). Crude model analysis gave an OR of 6.15 (95% CI: 1.57—24.13, p = 0.01) for endometriosis impact on ovarian cancer association which increased in Model 1 (OR: 6.96, 95% CI: 1.89—25.59, p = 0.004) and Model 2 (OR: 10.75, 95% CI: 2.74—42.22, p = 0.001). Crude model analysis also gave an OR of 3.97 (95% CI: 1.69—9.31, p = 0.002) for endometriosis impact on other cancers which remained significant after Model 1 (OR: 3.14, 95% CI: 1.30—7.56, p = 0.01) and Model 2 (OR: 2.80, 95% CI: 1.11—7.03, p = 0.03) analyses. No significant associations were found for breast, cervical, uterine, thyroid, skin or blood cancers (Table 4).

**Table 4:** Sensitivity analysis: weighted multivariable logistic regression analysis of the association between endometriosis and various types of cancer, with data after covariate imputation

Cancer	*n*	Crude model		Model 1		Model 2	
Type		OR (95%CI)	*p* value	OR (95%CI)	*p* value	OR (95%CI)	*p* value
Total cancer	240	2.31 (1.55, 3.43)	< 0.0001	1.81 (1.22, 2.71)	0.004	1.73 (1.13, 2.64)	0.01
Breast	36	1.32 (0.39, 4.53)	0.65	0.97 (0.28, 3.37)	0.97	0.93 (0.24, 3.53)	0.91
Cervix	79	1.79 (0.92, 3.52)	0.09	1.61 (0.80, 3.23)	0.18	1.30 (0.61, 2.75)	0.49
Uterus	20	0.90 (0.25, 3.22)	0.87	0.84 (0.23, 3.08)	0.79	1.07 (0.31, 3.76)	0.91
Ovary	14	6.15 (1.57, 24.13)	0.01	6.96 (1.89, 25.59)	0.004	10.75 (2.74, 42.22)	0.001
Thyroid	10	1.43 (0.15, 13.82)	0.75	1.08 (0.11, 10.37)	0.94	1.10 (0.11, 11.21)	0.93
Skin	32	2.50 (0.96, 6.50)	0.06	1.76 (0.66, 4.71)	0.25	1.79 (0.66, 4.88)	0.25
Blood	9	4.31 (0.88, 21.12)	0.07	3.20 (0.62, 16.47)	0.16	3.18 (0.53, 19.20)	0.20
Other	40	3.97 (1.69, 9.31)	0.002	3.14 (1.30, 7.56)	0.01	2.80 (1.11, 7.03)	0.03

Crude model: no adjustment;

Model 1: adjusted for age and race;

Model 2: adjusted for age, race, PIR, BMI, marital status, educational status, smoking, drinking, DM, hypertension;

*OR* odds ratios; *CI* confidence intervals

### MR

**Fig. 3:**
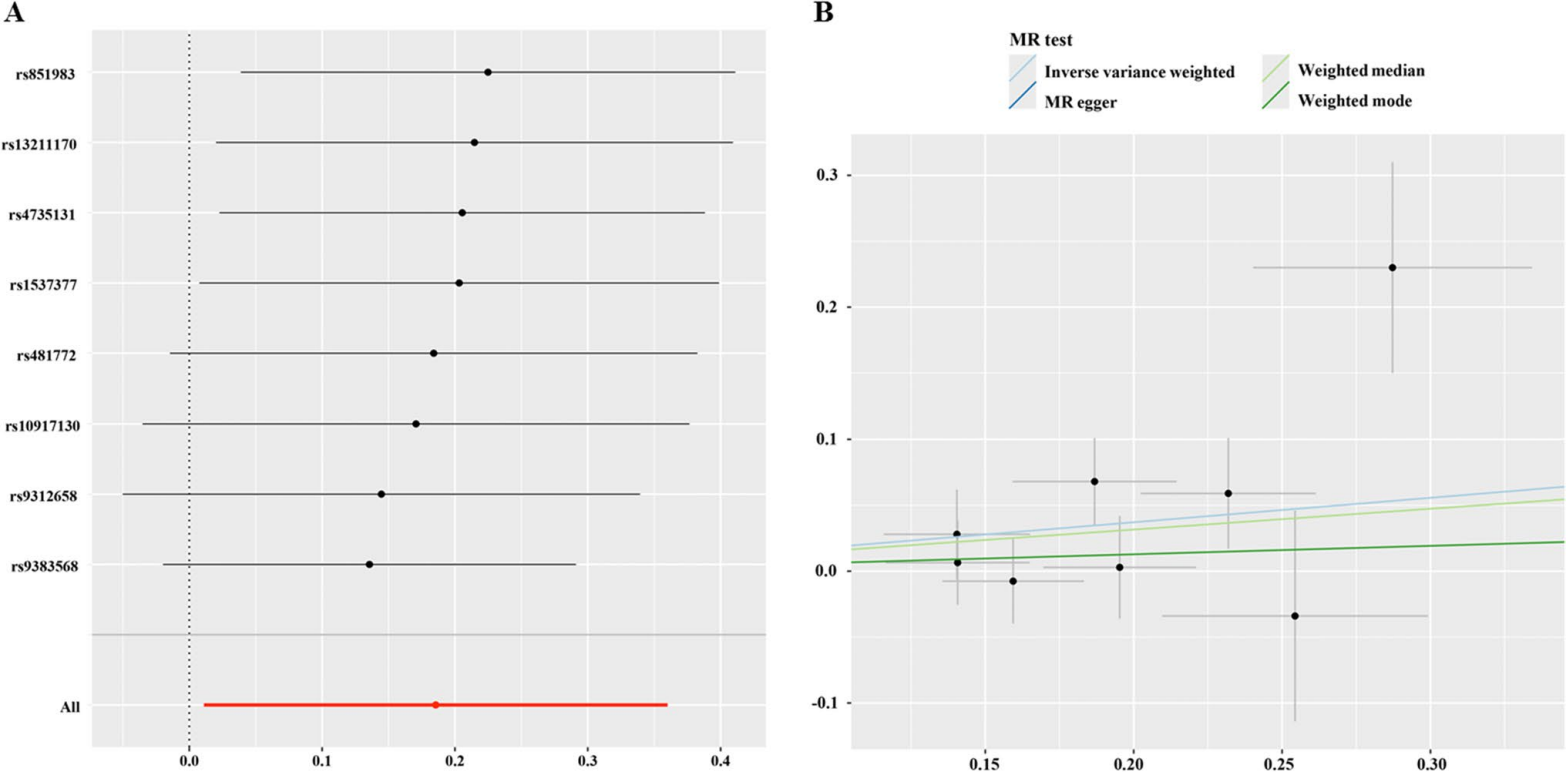
Mendelian Randomization (MR) analysis between endometriosis and ovarian cancer. **A**. Result of leave one out analysis; **B**. Scatter plot for four MR methods analysis

MR analysis with GWAS data from FinnGen and UK Biobank dataset supporting a positive association of endometriosis with ovarian cancer is presented in Table S1. Eight SNPs (rs851983, rs13211170, rs4735131, rs1537377, rs481772, rs10917130, rs9312658, and rs9383568) were found to support a positive association between endometriosis and ovarian cancer by IVW and leave-one-out analysis (Fig. 3A-B). IVW indicated that endometriosis was associated with an increased association of ovarian cancer (OR: 1.203, 95% CI: 1.011–1.433; p = 0.037). No significant heterogeneity was detected (Cochran’s Q test, p > 0.05). The MR-Egger regression intercept indicated no evidence of directional pleiotropy among the SNPs in the two datasets (p > 0.05). Comparative analyses using the weighted median and MR-PRESSO methods also demonstrated the risk effect of endometriosis on ovarian cancer, providing further evidence of the stability of the results obtained from the IVW method. These findings support the positive relationship between endometriosis and ovarian cancer.

## Discussion

The current analysis of the NHANES dataset represents a large-scale, population-based study of adults which found a significant high association between history of endometriosis and a high history of total cancer, especially in ovarian cancer, consistent with previous reports [30–32]. Women with history of endometriosis had an 11.40-fold association with history of ovarian cancer. In addition, MR analysis with GWAS data from FinnGen and UK Biobank showed evidence of a positive relationship between endometriosis and ovarian cancer. Women with endometriosis had lower overall survival and this was accounted for by mortality from non-cancer causes. The current analysis was conducted on a larger scale than previous evaluations of the links between endometriosis and ovarian cancer [30–35]. Such studies are valuable since both endometriosis and ovarian cancer remain underdiagnosed, stressing the urgent need to identify biomarkers to enable earlier diagnosis [8, 9, 36].

The links between endometriosis and cancer susceptibility are complex and are affected by demographic, environmental and genetic factors [37, 38]. Our study found that endometriosis is positively associated with overall cancer. However, when examining specific cancer types, we only observed a significant association with ovarian cancer, while no significant relationships were found with breast cancer, uterine cancer, or thyroid cancer. These findings differ from previous studies, such as a meta-analysis that reported a significantly increased risk of breast and thyroid cancers in women with endometriosis [33]. This discrepancy may be attributed to the relatively small sample size, limited follow-up duration, and small subgroup sizes for each cancer type in our study, which may have affected statistical power. Future research with larger sample sizes and longer follow-up periods is needed to further validate and analyze these associations. The outcome of the current work with an 11.40-fold prevalence of ovarian cancer in endometriosis participants than participants without endometriosis. Our study found a significant positive association between a history of endometriosis and a history of ovarian cancer, which is consistent with previous studies. A large cohort study in the United States found that women with ovarian endometriosis and/or deep infiltrating endometriosis had a 9.9-fold increased risk of ovarian cancer [39]. Similarly, a cohort study in Japan also indicated that women with ovarian endometriosis have an 8.95-fold higher risk of developing ovarian cancer compared to those without endometriosis [40]. However, other studies suggest that the risk is not as high. For example, a retrospective cohort study from California reported that women with endometriosis had a fourfold higher ovarian cancer incidence compared to those without endometriosis [41]. However, this study was based on a commercial insurance cohort, which may have introduced selection bias. Another meta-analysis showed that the risk of ovarian cancer in women with endometriosis was only increased by 1.93 times [33]. However, most of the studies included in this analysis had significant or critical risk of bias, and there was considerable heterogeneity across studies, especially regarding ovarian cancer, where publication bias was also present. This study found that the prevalence of endometriosis is significantly higher in White women compared to Black, Hispanic, and Mexican American women, suggesting that White individuals may have a stronger genetic susceptibility to endometriosis. A similar meta-analysis reached a comparable conclusion, indicating that Black women are less likely to develop endometriosis compared to White women [42]. However, the precise relationship between ethnicity, genetic susceptibility, and endometriosis remains unclear and warrants further investigation through genetic research.

An increased risk of mortality from all causes was found for the endometriosis cohort. This was particularly evident during the later phase of long-term follow-up beyond 165 months. Cancer-specific mortality was not different between participants with and without endometriosis, indicating that the difference in all-cause mortality between the two groups may be attributed to other diseases. It may be that autoimmune disorders have an impact in this respect [43, 44]. A Finnish endometriosis cohort had lower mortality rates, despite a high proportion of cancer-related deaths, suggesting that lifestyle factors or improved healthcare access might play protective roles [45]. The heterogeneity of findings in the literature emphasizes the complexity of the situation concerning endometriosis and illustrates that demographic, environmental, lifestyle and genetic factors are all influential. Future large-scale population-based cohort studies with long-term follow-up are needed to further clarify the association of endometriosis on cancer and mortality. Endometriosis is defined as the presence of endometrial-like tissue outside the uterus, inducing chronic inflammation and symptoms such as pelvic pain and infertility. This condition shares several characteristics with cancer, including tissue invasion, resistance to apoptosis, genetic alterations and activation of pathways like mTOR [46, 47]. GWAS have identified several loci common to both endometriosis and clear cell, endometrioid and serous ovarian cancer, indicating common pathophysiological pathways rather than isolated risk profiles for each condition [48, 49]. The chronic inflammation which characterizes endometriotic lesions, involving the secretion of pro-inflammatory cytokines and immune cells, promotes DNA damage, genomic instability and cell proliferation, creating a conducive environment for malignant transformation [50–52]. Moreover, autoimmune conditions and genetic mutations also in the pathophysiology of endometriosis and ovarian cancer. Studies have identified somatic point mutations, such as those in the estrogen receptor alpha (hERalpha), as important factors in adenomyosis and endometriosis, which may contribute to the malignant transformation of endometriotic tissue [53]. Additionally, mutations in steroid receptors, such as PROGINS, suggest that there may be a heritable, risk-increasing genetic background for these conditions [54]. Endometriosis therapy often consists of oral contraceptives, progestins and surgical interventions which may increase cancer risk. Accompanying conditions, such as infertility, anxiety and depression are also acknowledged to be cancer risk factors [55, 56]. An understanding of these factors and their interactions is vital to the improvement of care for women with endometriosis.

The strengths of this study include the use of representative NHANES data, with adjustment for various confounding factors. Additionally, we employed MR to explore the potential relationship between exposures and outcomes. This study also has limitations. First, this study relies on self-reported diagnostic data from NHANES, including diagnoses of endometriosis, cancer types, diabetes, and hypertension. These data may be subject to recall bias and inconsistencies in diagnostic criteria. Future studies should be based on clinical diagnoses from hospital systems to minimize these biases and improve diagnostic accuracy and consistency. Second, due to the relatively short follow-up period and the small number of total deaths, some cancer subgroups had limited cases, which may affect statistical power. In the future, extending the follow-up period by 5–10 years, with an increase in the number of death cases, would allow for further analysis to validate the findings. Third, since NHANES only recorded self-reported data on endometriosis between 1999 and 2006, the sample size is relatively small, and the data may not be sufficiently up-to-date. This could introduce selection bias. Fourth, although this study controlled for several confounding factors, residual confounding may still be present, particularly regarding factors such as hormone therapy use and number of pregnancies. Due to the limited data available in NHANES, these factors could not be adjusted for in the current analysis. Future studies should aim to account for these confounders to further refine the findings. Fifth, since this survey is a cross-sectional study, a temporal relationship cannot be established, and therefore, it cannot be used to infer future risk factors. As NHANES does not provide GWAS data, the MR analysis in this study used GWAS data from Finland and the UK Biobank dataset, which can only provide indirect evidence. Therefore, a direct causal relationship between endometriosis and ovarian cancer cannot be established. Sixth, the age, BMI, smoking, and alcohol consumption data used in this study are not based on the actual data at the time of endometriosis diagnosis, but rather on baseline data collected during the survey. However, these variables exhibit predictable patterns: age tends to increase over time, smoking and alcohol consumption are long-term habits, and BMI remains relatively stable over time. Therefore, these data were used for statistical analysis. Although the results may not directly reflect the conditions at the time of diagnosis, they provide indirect insights and may be subject to some bias. Future studies should analyze demographic data, lifestyle habits, and comorbidities at the time of endometriosis diagnosis to further validate our findings. Finally, the cross-sectional data used in this study were obtained from the NHANES conducted in the United States. The GWAS data used in our MR analysis were from FinnGen and UK Biobank dataset. MR analyses rely primarily on genetic data, they are limited in accounting for non-inherited factors. Given the differences in geographical environment, lifestyle habits, and genetic susceptibilities across countries and regions, the applicability of our MR results to the American or other populations is limited as the influence of epigenetic modifications is not accounted for.

## Conclusions

In conclusion, our findings suggest a positive correlation between history of endometriosis and history of ovarian cancer. Given the limitations of this study, future research based on large cohort studies, utilizing clinical diagnosis data and more comprehensive GWAS data, is needed to further explore this relationship.

## Supplementary Information

Below is the link to the electronic supplementary material.

Supplementary file1 (DOCX 19 KB)

## Data Availability

The NHANES dataset is publicly available online, accessible at https://wwwn.cdc.gov/nchs/nhanes/default.aspx
