# Supplementary material for: Endometriosis and Ovarian Cancer: Insights from NHANES and Mendelian Randomization Analysis
Source: Reprod Sci. 2025 Jun 23;32(7):2283–94. doi: 10.1007/s43032-025-01910-x (PMC12270951; doi:10.1007/s43032-025-01910-x)
Supplement: Supplementary file 1 — Supplementary file1 (DOCX 19 KB) [file 43032_2025_1910_MOESM1_ESM.docx]

**Supplementary table**

Table S1. MR analysis data supporting a causal effect of endometriosis on ovarian cancer

| **Outcome** | **Exposure** | **Method** | **nsnp** | **b** | **se** | **pval** | **lo_ci** | **up_ci** | **or** | **or_lci95** | **or_uci95** |
| --- | --- | --- | --- | --- | --- | --- | --- | --- | --- | --- | --- |
| Malignant neoplasm of ovary \|\| id:ukb-saige-184.11 | Endometriosis diagnosis and infertility diagnosis occurring together \|\| id:finngen_R10_N14_ENDOMET_INFERT | Inverse variance weighted | 8 | 0.186 | 0.089 | 0.037 | 0.011 | 0.360 | 1.204 | 1.011 | 1.433 |
| Malignant neoplasm of ovary \|\| id:ukb-saige-184.11 | Endometriosis diagnosis and infertility diagnosis occurring together \|\| id:finngen_R10_N14_ENDOMET_INFERT | MR Egger | 8 | 0.675 | 0.409 | 0.150 | -0.126 | 1.477 | 1.965 | 0.881 | 4.380 |
| Malignant neoplasm of ovary \|\| id:ukb-saige-184.11 | Endometriosis diagnosis and infertility diagnosis occurring together \|\| id:finngen_R10_N14_ENDOMET_INFERT | Weighted median | 8 | 0.158 | 0.104 | 0.131 | -0.047 | 0.362 | 1.171 | 0.954 | 1.437 |
| Malignant neoplasm of ovary \|\| id:ukb-saige-184.11 | Endometriosis diagnosis and infertility diagnosis occurring together \|\| id:finngen_R10_N14_ENDOMET_INFERT | Weighted mode | 8 | 0.064 | 0.155 | 0.692 | -0.240 | 0.368 | 1.066 | 0.787 | 1.444 |

Abbreviations: nsnp: Number of SNPs; b: Coefficient; se: Standard Error; pval: p-value; lo_ci: Lower Confidence Interval; up_ci: Upper Confidence Interval; or: Odds Ratio; or_lci95: 95% Lower Confidence Interval of Odds Ratio; or_uci95: 95% Upper Confidence Interval of Odds Ratio.
